# Diseases of the Nervous System

**Published:** 1894-12-15

**Authors:** 


### Progress in Medicine.

### DISEASES OF THE NERVOUS SYSTEM.

(*Continued from page* 174.)

**Speech Centres.**—Ord and Shattock[25] describe the left hemisphere from a case of aphasia. There was an area of softening in the lower frontal convolution—the proper aphasic area of Broca; and behind this another area occupying the lowest part of the ascending frontal convolution; this area extended in the upward direction from the posterior limb of the Sylvian fissure for one inch, involving the larger anterior part of the convolution. In life the only facial affection was some paralysis of the opposite levator labii superioris. It would seem that although the face and tongue centre lay in the lowest third of the ascending frontal convolution, there was a special area in the anterior portion of the lowest third of this convolution,, which was not part of this centre. Semon and Horsley had shown this area to be the phonatory centre in monkeys, and the authors thought it might provisionally be held to have the same function in man. The centre in each hemisphere has apparently however a bilateral function.

**Reflex ocular neuroses.**—This subject continues to be the cause of much debate. Weir Mitchell[26] states that no notable cure has resulted in 100 cases of epilepsy and chorea by correction of refractive or other ocular errors, and as regards the efficacy of tenotomy and adjustment of muscular anomalies his experience is negative. F. H. Bartlett[27] while by no means agreeing with the view that correction of ocular defects will procure relief for every neurotic case which presents them, still on the whole strongly recommends this line of treatment in conjunction with other more familiar methods. Casey A. Wood,[28] in a long review of the question of the treatment of epilepsy by tenotomy of the eye muscles, arrives at a series of important conclusions. (1) Practically everybody has heterophoria, and therefore epileptics may be expected to have heterophoria also. (2) It is admitted that the correction of refractive errors has cured idiopathic epilepsy. (3) When an epileptic is unaware of any defect of vision and has practically normal eyes, eye treatment is not indicated in the case. (4) The effect of impressions upon the nervous system of an epileptic by an operation, coupled with the hope held out of relief by a new surgical procedure, must be taken into consideration; often it is probable that some other operation than graduated tenotomy would have been as effectual in relieving the epileptic tendency. (5) It remains to be shown that anomalies of the extrinsic eye-muscles are responsible for the seizures of epilepsy. (6) The eyes should be carefully examined in every case of epilepsy where asthenopic symptoms are present. Joseph Collins[29] gives it as his opinion that the eye-plan of treatment of epileptics, even when looked at with a most charitable eye, does not deserve the laudations of most of its advocates; it can never be more than a factor in the treatment of the disease.

**Epilepsy.**—Flechsig's plan of treatment is recorded by Dr. Collins in the paper just quoted. The method consists in administering opium in large doses for a period of six weeks. The dose of opium in the beginning is from one-half to one grain, gradually increased until the patient is taking fifteen grains or more per day, the maximum dose being attained by the end of the first week. At the end of six weeks the opium is suddenly stopped, and for it bromide of potassium or sodium in doses of thirty grains four times a day is substituted. After these large doses of bromide have been kept up for some time the dose is gradually decreased until less than forty grains is taken in one day. The sudden cessation of administering the opium and administering the bromide is essential. The most satisfactory results are obtained in very chronic epileptics, and in epilepsy depending upon gross organic lesions the treatment gives better results than in pure idiopathic epilepsy. The plan is particularly valuable in old intractable cases, but Flechsig's treatment is not a specific, for although in almost every case so treated mprovement has followed, a relapse generally occurs in the course of a few weeks or months, though the relapse is less severe in type than the original malady. No diminution of the frequency of the fits is seen during the period of the opium taking, which simply enhances the effect of the bromides administered subsequently. Care is necessary in administration of the opium at first, until some tolerance is established.

**Hysteria.**—The close simulation of organic disease by hysterical conditions is becoming increasingly recognised. Comby[30] records the case of a woman who, three times under the influence of thundery weather had sustained sudden attacks of "apoplexy," *i.e.*, unconsciousness followed by hemiplegia and hemi-anæsthesia. Recovery had been complete on each occasion. Bischoff[31] also records a case of a man suddenly becoming hemiplegic and hemi-anæsthetic upon the left side, being at the same time quite aphasic with contraction of the field of vision; phonation, though not articulation, was preserved; there was no trace of spasm of the face muscles. Recovery was ultimately complete. Diller[32] describes the case of a man who suffered from frequently recurring attacks of complete motor aphasia, lasting several hours, consciousness and the ability to read and write being preserved throughout. Later he became subject to attacks of lethargy, in which he would lie "like dead" for two or three hours, and finally recovered completely under hypnotism and suggestion. Hirt[33] states that he has seen three cases of general muscular wasting in women due to hysteria, but all fatal. The one case recorded in detail occurred in a girl in an epidemic of hysterical spasm in a school. R.D. was absent, and there was no sign of organic disease, such as diabetes or tuberculosis, &c., being present. The case ended fatally. Charcot[34] speaks of a case of amyotrophic lateral sclerosis or of hysterical muscular wasting. He was unable to decide to which class the case should be assigned. Schwab[35] reviews the condition described by Charcot as traumatic hystero-neurasthenia, frequently seen after railway injuries and so forth. The course of the disease is very variable, and prognosis differs largely according to the observer. If besides such symptoms as could be simulated, there are added contraction of the visual field, cardio-vascular symptoms, fibrillary twitchings, exaggerated reflexes, gastro-intestinal atony and impairment of general health, the idea of simulation may be absolutely rejected. Effertz[36] showed in New York a woman who, for sixteen years, had had contraction of the hands of both upper extremities, and complete anæsthesia of the face and body; under hypnotism the contracture was temporarily relaxed, but the anæsthesia remained uninfluenced. J. M. Clarke[37] gives a very lengthened *résumé* of recent literature upon hysteria. Speaking generally, this excellent report impresses one forcibly with the portentously increased area which is now included within the diagnosis of hysteria, largely different from what has obtained until the last few years.

[25] Brit. Med. Journ., March 24, 1894. [26] Philade'phia Med. News, quoted in Lancet, June 2, 1894. [27] New York Med. Journ., June 16, 1894. [28] New York Med. Journ., July 7 and 14, 1894. [29] Medical Record, Sept. 22, 1894. [30] Med. Week, June 1, 1894. [31] Wien. Med. Woch., quoted in Epitome Brit. Med. Journ., May 26, 1894. [32] Internat. Med. Mag., April, 1894. [33] Deut. Med. Woch., quoted in Ep. Brit. Med. Journ. June 23, 1894. [34] Fortschrit der Med., No. 7, 1894. [35] Medicine Moderne, quoted Med. Rec., May 12, 1894. [36] New York Med. Journ., June 2, 1894. [37] Brain, Parts LXV. and LXVI.

